# Circulating memory B cells and plasmablasts are associated with the levels of serum immunoglobulin in patients with ulcerative colitis

**DOI:** 10.1111/jcmm.12728

**Published:** 2016-01-22

**Authors:** Xinrui Wang, Yanfang Jiang, Yonggang Zhu, Manli Zhang, Man Li, Hongjuan Wang, Pujun Gao

**Affiliations:** ^1^ Department of Central LaboratoryThe First HospitalJilin UniversityChangchunChina; ^2^ Key Laboratory of Zoonosis ResearchMinistry of EducationThe First HospitalJilin UniversityChangchunChina; ^3^ Jiangsu Co‐innovation Center for Prevention and Control of Important Animal Infectious Diseases and ZoonosesYangzhouChina; ^4^ Department of Radiation OncologyChina‐Japan Union Hospital of Jilin UniversityChangchunChina

**Keywords:** ulcerative colitis, memory B cells, plasmablasts, IgG

## Abstract

Humoural immunity is crucial for the pathogenesis of ulcerative colitis (UC), but the precise perturbation of B cell immunity is poorly understood. This study is aimed at evaluating the numbers of different subsets of circulating memory B cells, plasmablasts, and the levels of serum immunoglobulin in UC patients. Total of 23 patients with active UC and 14 healthy controls (HC) were examined for the numbers of different subsets of circulating memory B cells and plasmablasts before and after treatment with mesalazine for 8–12 weeks by flow cytometry. Disease activity was evaluated by the Mayo clinic score. The levels of serum immunoglobulin, C‐reactive protein (CRP) and erythrocyte sedimentation rate (ESR) were measured in individual subjects. In comparison with that in HC, significantly reduced numbers of IgG^+^ IgD^−^ CD27^+^ CD19^+^ memory B cells, increased numbers of CD20^−^ CD19^+^ plasmablast subsets, and higher serum IgG levels were detected in UC patients. The concentrations of serum IgG, the numbers of CD138^+^ CD38^+^ CD20^−^ CD19^+^, and IgG^+^ CD38^+^ CD20^−^ CD19^+^ plasmablasts were negatively associated with the numbers of IgG^+^ IgD^−^ CD27^+^ CD19^+^ memory B cells. Furthermore, the values of Mayo clinic score, CRP, or ESR in UC patients were negatively correlated with the numbers of IgG^+^ IgD^−^ CD27^+^ CD19^+^ memory B cells, while positively correlated with the serum IgG levels and the numbers of plasmablast subsets. Following treatment with mesalazine, the numbers of circulating IgG^+^ IgD^−^ CD27^+^ CD19^+^ memory B cells were significantly increased, while the numbers of CD138^+^ CD38^+^ CD20^−^ CD19^+^ and IgG^+^ CD38^+^ CD20^−^ CD19^+^ plasmablasts were reduced in UC patients. These decreased IgG^+^ IgD^−^ CD27^+^ CD19^+^ memory B cells and increased plasmablasts may be involved in the pathogenic process of UC.

## Introduction

Ulcerative colitis (UC) is an idiopathic inflammatory bowel disease (IBD). Ulcerative colitis has a relapsing and remitting course and is characterized by the chronic inflammation in the rectum and colon 1, 2. Although the precise aetiology and the pathogenic process of UC remains unclear, interaction of genetic with environmental factors results in a dysregulated immune response in the gut, contributing to the pathogenesis of UC 3, 4, 5. Hyperactive T cells and their excessive production of pro‐inflammatory cytokines are the major immunological factors 6. However, the role of B cells and plasma cells in the pathogenesis of UC has not been clarified 5, 7, 8.

Blood circulating B cells have long been identified as CD3^−^ CD19^+^ cells 9, including IgD^+^ CD27^−^ naïve B cells, IgD^+^ CD27^+^ non‐class‐switched memory B cells, and IgD^−^ CD27^+^ class‐switched memory B cells 10, 11. Classically, the IgD^−^ CD27^+^ memory B cells have switched their IgM to IgG, IgA or IgE 10, besides a new subset of IgM‐expressing memory B cells 12, 13. It is believed that IgG^+^ memory B cells are the frontline responders by directly giving rise to IgG‐secreting cells, while IgM^+^ memory cells return to the germinal centre 14. Previous studies have shown that there is no significant difference in the frequency of class‐switched memory B cells between UC patients and healthy individuals 15, 16. However, there is little information about the frequency of circulating IgG^+^ and IgM^+^ class‐switched memory B cells in patients with UC.

Upon activation, naïve and memory B cells can differentiate into plasma cells, which produce antibodies. Circulating plasmablasts are proliferating, immature precursors of tissue plasma cells, which express low or little CD20 17, 18. Several studies have found significant increased numbers of plasma cells or plasmablasts in UC patients compared with healthy controls (HC) 19, 20, 21, and B cells and IgA+ or IgG+ plasma cells were detected in the lesions of UC patients 22, 23. However, little information is available about the numbers of different subsets of circulating plasmablasts and the levels of serum Ig in patients with UC as well as the potential relationship between the numbers of plasma cells and memory B cells.

In this study, we characterized the numbers of different subsets of circulating memory B cells and plasmablasts, and the levels of serum immunoglobulin in active UC patients and HC. We discussed the implications of our findings.

## Materials and methods

### Patients and controls

A total of 23 patients with active UC were recruited at the inpatient service of the Department of Gastroenterology, the First Hospital of Jilin University (Changchun, China) from July 2014 to March 2015. Another 14 gender‐, age‐ and ethnicity‐matched HC were recruited from the Physical Examination Center of our hospital during the same period, and these HC had no known autoimmune diseases, inflammatory diseases, or allergies. Individual patients with UC were diagnosed on the standard criteria using clinical, radiographic, endoscopic and histological findings 4. Patients with indeterminate colitis, any other inflammatory disease, or those who had received conventional corticosteroids, immune‐suppressive drugs or targeted biological therapy within the past 3 months were excluded from this study 24. The disease activity of individual patients was evaluated by the Mayo Clinic score 25. A UC patients with a Mayo Clinic score of >3 was considered as active. Written informed consent was obtained from individual subjects. The experimental protocol was established according to the guidelines of the Declaration of Helsinki and was approved by the Human Ethics Committee of Jilin University. The demographic and clinical characteristics of individual participants are shown in Table 1.

**Table 1:** The demographic and clinical characteristics of participants

Parameters	UC	HC
No.	23	14
Age (years)	48 (15–74)	49 (19–68)
Gender: female/male	9/14	5/9
WBC (10^9^/l)	7.19 (3.36–12.56)	6.43 (4.92–9.21)
Lymphocytes (10^9^/l)	1.95 (0.87–3.39)	1.68 (0.50–2.72)
ESR (mm/hr)	36.8 (5–95)^a^	3.5 (1.5–16)
CRP (mg/l)	30.7 (1.7–163)^a^	1.8 (1.3–3.6)
Haemoglobin (g/l)	107 (85–150)^a^	145 (126–180)
Hematocrit (%)	34.8 (27.0–44.3)^a^	45.6 (39.1–52.0)
Serum albumin (g/l)	34.96 (20.58–42.80)^a^	44.20 (40.65–49.11)
Mayo clinic score	7 (3–12)	NA

a *P* < 0.05 *versus* the HC.

Data are median (range) or real case number.

Normal values: WBC: 3.50–9.50 (10^9^/l), Lymphocytes: 1.10–3.20 (10^9^/l), ESR: 0–15 (mm/hr), CRP: 0–3 (mg/l), Haemoglobin: 130–175 (g/l), Hematocrit: 40.0–50.0 (%), and Serum albumin: 40.00–55.00 (g/l).

UC: ulcerative colitis; HC: healthy control; WBC: White blood cell counts; ESR: Erythrocyte sedimentation rate; CRP: C‐reactive protein; NA: Not available.

### Treatment and follow‐up

Individual patients were treated orally with mesalazine (1500 mg/day; Losan Pharma GmbH, Neuenburg, Germany) or a combination of oral and topical preparations of mesalazine, along with montmorillonite powder (Qianjin Xiangjiang Pharmaceutical, Hunan, China) to relieve diarrhoea. The patients were followed up for 8–12 weeks. There were altogether six patients with complete records and the other 17 patients failed to follow‐up. Blood samples were collected before and 8–12 weeks after the treatment.

### Clinical examination

The clinical data of each subject were collected from the hospital records. These data included age, gender and laboratory tests. Individual subjects were subjected to routine laboratory tests for full blood cell counts, erythrocyte sedimentation rate (ESR), the concentrations of serum C‐reactive protein (CRP) and albumin using ADVIA 1650 biochemical analyzer (Bayer, Pittsburg, PA, USA).

### Flow cytometry analysis

Fasting venous blood samples were collected from individual subjects, and peripheral blood mononuclear cells were isolated by density‐gradient centrifugation using Ficoll‐Paque Plus (Amersham Biosciences, Little Chalfont, UK). Peripheral blood mononuclear cells at 1 × 10^6^/tube were stained in duplicate with APC‐H7‐anti‐CD3, PerCP‐Cy5.5‐anti‐CD19, PE‐Cy7‐anti‐CD27, APC‐anti‐CD38, PE‐anti‐CD138, PE‐CF594‐anti‐CD20, FITC‐anti‐IgD (BD Biosciences, San Jose, CA, USA) in the dark at room temperature for 30 min. After being washed, the cells were fixed and permeabilized using a fixation/permeabilization kit (BD Biosciences), followed by intracellular staining with BV510‐anti‐IgG, and BV421‐anti‐IgM (BD Biosciences). Negative controls were stained with isotype‐matched control antibodies (APC‐H7‐anti‐IgG1, PerCP‐Cy5.5‐anti‐IgG1, PE‐Cy7‐anti‐IgG1, APC‐anti‐IgG1, PE‐anti‐IgG1, PE‐CF594‐anti‐IgG2b, FITC‐anti‐IgG2a, BV421‐anti‐IgG1, BV510‐anti‐IgG1; BD Biosciences). The percentages of different subsets of B cells were characterized on a FACSAria II (Becton Dickinson, San Jose, CA, USA) and at least 50,000 events were analysed by FlowJo software (v5.7.2; TreeStar Inc., Ashland, OR, USA). The number of each type of cells tested was calculated, according to the percentages of this type of cells multiplied lymphocyte counts.

### Cytometric Bead Array analysis of serum immunoglobulin

The concentrations of serum total IgG, IgM and IgA were determined by Cytometric Bead Array (CBA) 26, according to the manufacturer's protocol (BD Biosciences) with minor modifications. Briefly, for the measurement of serum total IgG, individual sera (5 μl/each) were diluted to the final dilution 1:62,500 and tested in duplicate. For the measurement of serum IgM and IgA, individual sera (6 μl/each) were diluted to the final dilution 1:10,648 and tested in duplicate. The concentrations of serum immunoglobulin were quantified using the CellQuest Pro and CBA software (Becton Dickinson) on a FACSAria II.

### Statistical analysis

Data are expressed as median and range. The difference between two groups was analysed by the Mann–Whitney *U* non‐parametric test. The difference between pre‐treatment and post‐treatment patients was analysed using the Wilcoxon test. The relationship between variables was evaluated using the Spearman rank correlation test. The raw P values of correlation analysis were adjusted by the Benjamini and Hochberg (BH) correction procedure to account for multiple tests with FDR <5% 27. The correction of raw *P*‐values was performed using the *P* adjust procedure in R language, and all the *P*‐values of correlation analysis mentioned in the result section were adjusted *P*‐values. All the statistical analyses except the BH correction were performed by the SPSS version 19.0 software (IBM, Armonk, New York, USA). A two‐sided *P* < 0.05 was considered statistically significant.

## Results

### Patient characteristics

There was no significant difference in the distribution of age and gender as well as in the numbers of white blood cells and lymphocytes between the UC patients and HC (Table 1). The values of ESR and CRP were significantly higher in the patients than the HC, but the values of haemoglobin, hematocrit and serum albumin were significantly less in the patients than in the HC, suggesting that the patients had inflammation and in the state of anaemia and mal‐nutrition because of chronic bleeding. In addition, UC patients displayed variable values of Mayo clinic score.

### Numbers of circulating naïve and memory B cells in UC patients

**Figure 1:**
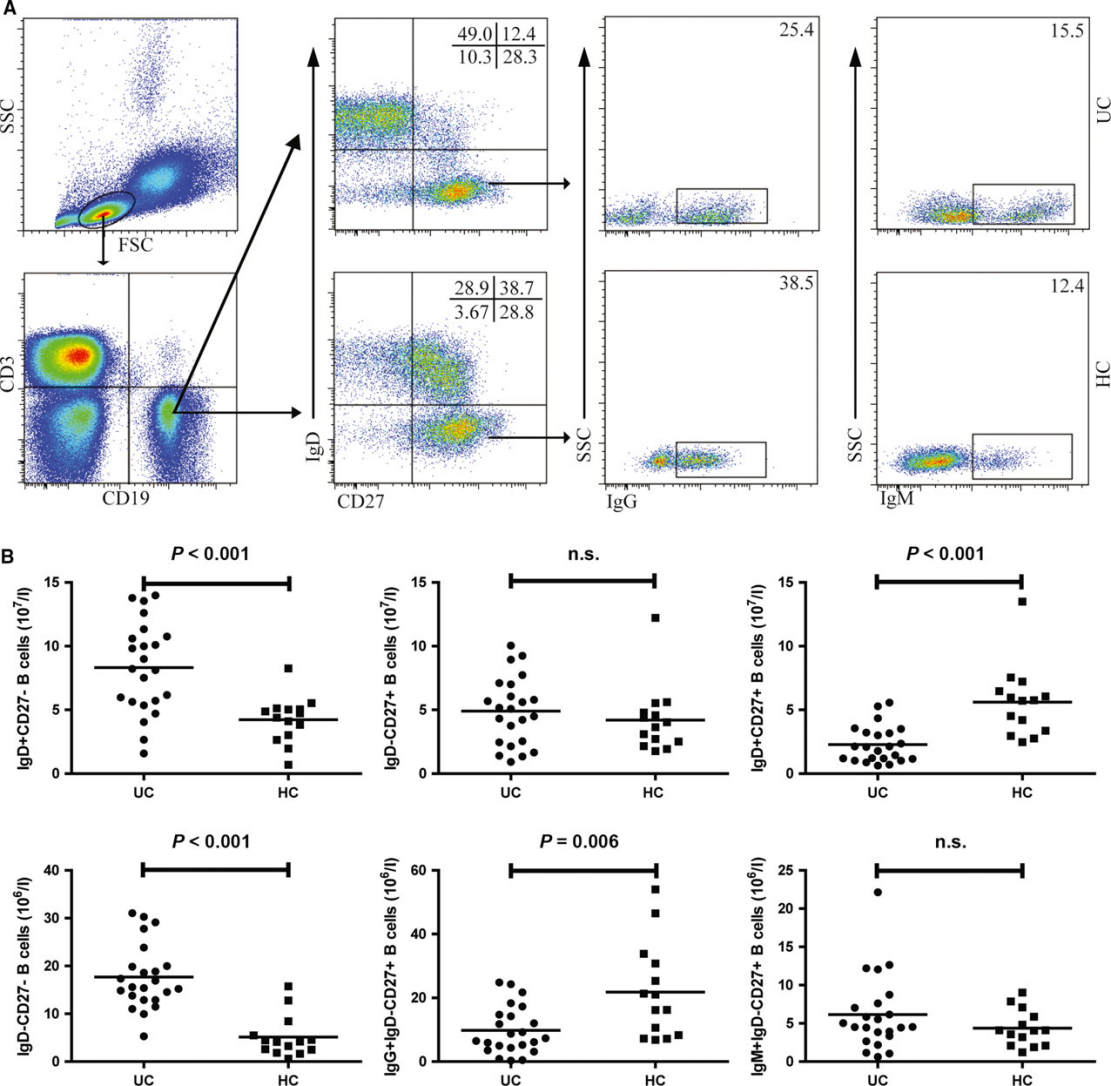
Flow cytometry analysis of the numbers of circulating B cells in UC patients. Peripheral blood mononuclear cells (PBMCs) from UC patients and HC were stained with fluorescent anti‐CD3, anti‐CD19, anti‐CD27, anti‐IgD, anti‐IgG and anti‐IgM. The cells were gated initially on living lymphocytes, and on CD19^+^ CD3^−^ B cells and then on IgD^−^ CD27^+^ B cells. The numbers of IgD^+^ CD27^−^, IgD^+^ CD27^+^, IgD^−^ CD27^+^, IgD^−^ CD27^−^, IgG^+^ IgD^−^ CD27^+^ and IgM^+^ IgD^−^ CD27^+^ B cells were analysed by flow cytometry. (**A**) Flow cytometry analysis. (**B**) Quantitative analysis. Data shown are representative dot plug or expressed as the mean% B cells of individual subjects. The difference between two groups was analysed by the Mann–Whitney *U* nonparametric test. The horizontal lines represent the median values.

We characterized the numbers of peripheral IgD^+^ CD27^−^ naïve B cells, IgD^+^ CD27^+^ non‐class‐switched memory B cells and IgD^−^ CD27^+^ class‐switched memory B cells between the UC patients and HC by flow cytometry. As shown in Figure 1, the numbers of IgD^+^ CD27^−^ naïve B cells in those patients were significantly greater than that in the HC (*P* < 0.001). In contrast, the numbers of IgD^+^ CD27^+^ non‐class‐switched memory B cells were significantly less in the patients than that in the HC (*P* < 0.001). There was no significant difference in the numbers of IgD^−^ CD27  class‐switched memory B cells between the UC patients and HC. The numbers of IgD^−^ CD27^−^ double‐negative B cells in UC patients were significantly increased compared with the HC (*P* < 0.001). Given different roles of IgM^+^ class‐switched memory B cells and IgG^+^ class‐switched memory B cells, we further analysed the numbers of IgM^+^ IgD^−^ CD27^+^ and IgG^+^ IgD^−^ CD27^+^ class‐switched memory B cells. Our data indicated the numbers of IgG^+^ IgD^−^ CD27^+^ class‐switched B cells in the patients were significantly less than that in the HC (*P* = 0.006), while there was no significant difference in the numbers of IgM^+^ IgD^−^ CD27^+^ class‐switched B cells between the UC patients and HC.

### Numbers of circulating CD20^−^ CD19^+^ plasmablasts in UC patients

**Figure 2:**
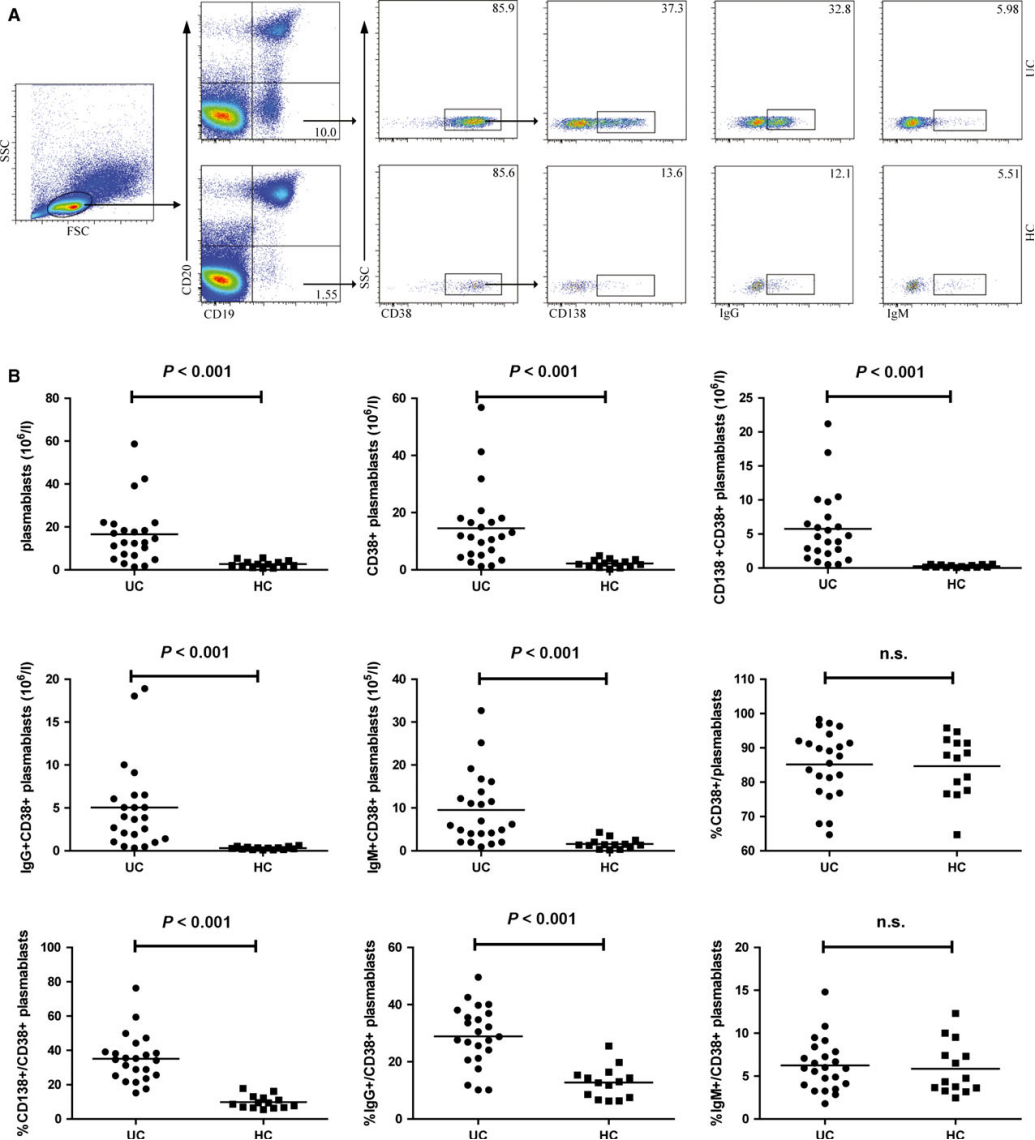
Flow cytometry analysis of the numbers of circulating CD138^+^ or IgG^+^ plasmablasts in UC patients. Peripheral blood mononuclear cells (PBMCs) from UC patients and HC were stained with fluorescent anti‐CD19, anti‐CD20, anti‐CD38, anti‐CD138, anti‐IgG and anti‐IgM. The cells were gated initially on living lymphocytes, and then on CD20^−^ CD19^+^ plasmablasts, and subsequently on CD38^+^ CD20^−^ CD19^+^ plasmablasts, and CD138^+^ CD38^+^ CD20^−^ CD19^+^ plasmablasts. The numbers of CD20^−^ CD19^+^, CD38^+^ CD20^−^ CD19^+^, CD138^+^ CD38^+^ CD20^−^ CD19^+^, IgG^+^ CD38^+^ CD20^−^ CD19^+^ and IgM^+^ CD38^+^ CD20^−^ CD19^+^ plasmablasts was analysed by flow cytometry. (**A**) Flow cytometry analysis. (**B**) Quantitative analysis. Data shown are representative dot plug or expressed as the mean% plasmablasts of individual subjects. The difference between two groups was analysed by the Mann–Whitney *U* nonparametric test. The horizontal lines represent the median values.

Next, we analysed the numbers of circulating CD20 ^−^CD19^+^ plasmablasts. As shown in Figure 2, many CD20^−^ CD19^+^ cells were CD38^+^, a marker of plasma cells. The numbers of CD20^−^ CD19^+^ plasmablasts in the patients were significantly greater than that in the HC (*P* < 0.001). Furthermore, the numbers of CD38^+^, CD138^+^ CD38^+^, IgG^+^ CD38^+^ and IgM^+^ CD38^+^ plasmablasts were also significantly greater than that in the HC (*P* < 0.001, *P* < 0.001, *P* < 0.001, *P* < 0.001). We analysed the percentages of different subsets of plasmablasts and found that the percentages of circulating CD138^+^ CD38^+^ and IgG^+^ CD38^+^ plasmablasts in the UC patients were significantly higher than that in the HC (*P* < 0.001, *P* < 0.001). However, there was no significant difference in the percentages of IgM^+^ CD38^+^ plasmablasts between the UC patients and HC, although the numbers of these cells were increased in UC patients.

### The relationship between the numbers of IgG^+^ IgD^−^ CD27^+^ CD19^+^ memory B cells or CD20^−^ CD19^+^ plasmablasts and the values of clinical parameters in UC patients

**Figure 3:**
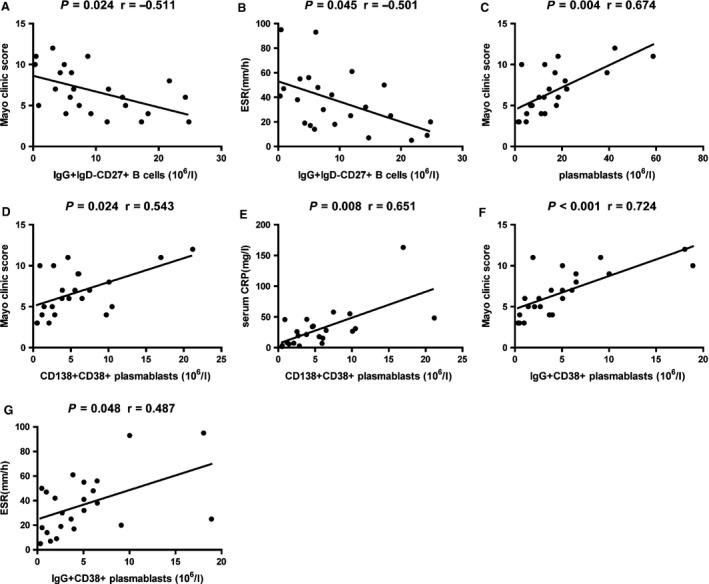
Correlation analysis. The potential correlations among the numbers of different subsets of B cells, plasmablasts and the values of clinical parameters were analysed by the Spearman correlation tests. BH correction was performed for multiple correlation. (**A** and **B**) The values of Mayo clinic scores, and ESR were negatively correlated with the numbers of IgG^+^ IgD^−^ CD27^+^ memory B cells. (**C**) The values of Mayo clinic scores were positively correlated with the numbers of plasmablasts. (**D** and **E**) The values of Mayo clinic scores, and the concentrations of serum CRP were positively correlated with the numbers of CD138^+^ CD38^+^ plasmablasts. (**F** and **G**) The values of Mayo clinic scores, and ESR were positively correlated with the numbers of IgG^+^ CD38^+^ plasmablasts.

To understand the importance of different subsets of memory B cells and plasmablasts in the pathogenesis of UC, we analysed the potential association of the numbers of different subsets of circulating B cells with the values of clinical measures tested in these patients. As shown in Figure 3, we found that the values of Mayo clinic score and ESR were negatively correlated with the numbers of IgG^+^ IgD^−^ CD27^+^ memory B cells (*P* = 0.024, *r* = −0.511; *P* = 0.045, *r* = −0.501). There was no significant association between the values of Mayo clinic scores, ESR or CRP and the numbers of other subsets of naïve and memory B cells (data not shown). Furthermore, the Mayo clinic scores were positively correlated with the numbers of CD20^−^ CD19^+^ plasmablasts (*P* = 0.004, *r* = 0.674). However, there was no significant association among the values of ESR, the concentrations of CRP and the numbers of CD20^−^ CD19^+^ plasmablasts (data not shown). Further analysis revealed that the Mayo clinic scores and the concentrations of CRP were positively correlated with the numbers of CD138^+^ CD38^+^ plasmablasts (*P* = 0.024, *r* = 0.543; *P* = 0.008, *r* = 0.651). In addition, the values of Mayo clinic score and ESR were positively correlated with the numbers of IgG^+^ CD38^+^ plasmablasts (*P* < 0.001, *r* = 0.724; *P* = 0.048, *r* = 0.487). These data suggest that IgG^+^ IgD^−^ CD27^+^ CD19^+^ memory B cells and different subsets of CD20^−^ CD19^+^ plasmablasts may have different functions in the pathogenesis of UC.

### The relationship between the numbers of circulating IgG^+^ IgD^−^ CD27^+^ CD19^+^ memory B cells and different subsets of CD20^−^ CD19^+^ plasmablasts in UC patients

**Figure 4:**
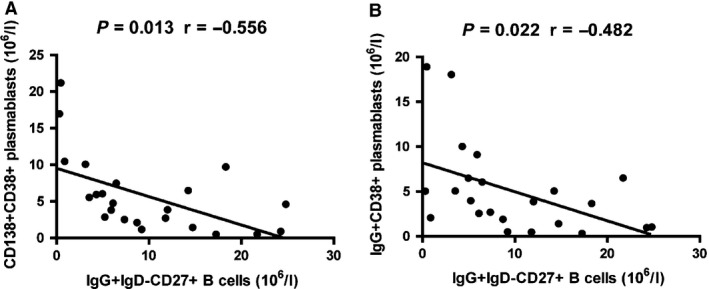
Correlation analysis. The potential correlations among the numbers of different subsets of B cells and plasmablasts were analysed by the Spearman correlation tests. BH correction was performed for multiple correlation. (**A** and **B**) The numbers of CD138^+^ CD38^+^ and IgG^+^ CD38^+^ plasmablasts were negatively associated with the numbers of IgG^+^ IgD^−^ CD27^+^ memory B cells, respectively.

Initially, we analysed the potential relationship between the numbers of circulating IgG^+^ IgD^−^ CD27^+^ CD19^+^ B cells with different subsets of CD20^−^ CD19^+^ plasmablasts in the UC patients. There was no significant association between the numbers of CD20^−^ CD19^+^ plasmablasts and the numbers of IgG^+^ IgD^−^ CD27^+^ CD19^+^ memory B cells in the UC patients (data not shown). However, the numbers of CD138^+^ CD38^+^ CD20^−^ CD19^+^ and IgG^+^ CD38^+^ CD20^−^ CD19^+^ plasmablasts were correlated negatively with the numbers of IgG^+^ IgD^−^ CD27^+^ CD19^+^ memory B cells in the UC patients (*P* = 0.013, *r* = −0.556; *P* = 0.022, *r* = −0.482, Fig. 4). Our data indicated that IgG^+^ IgD^−^ CD27^+^ CD19^+^ memory B cells and different subsets of CD20^−^ CD19^+^ plasmablasts contributed to the pathogenesis of UC.

### Correlation analysis of clinic pathological features of UC with the concentrations of IgG, IgM and IgA

**Figure 5:**
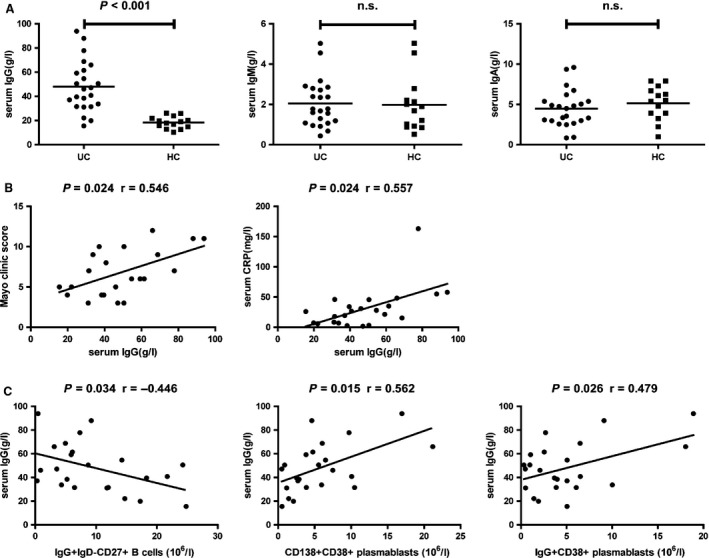
The levels of serum IgG, IgM and IgA, and their correlation with clinic pathological features in UC patients. (**A**) The levels of serum IgG, IgM and IgA in individual subjects were tested by CBA. (**B**) The values of Mayo clinic scores and the concentrations of serum CRP were positively correlated with the concentrations of IgG in UC patients. (**C**) The concentrations of IgG were negatively associated with the numbers of IgG^+^ IgD^−^ CD27^+^ memory B cells, and positively associated with the numbers of CD138^+^ CD38^+^ and IgG^+^ CD38^+^ plasmablasts in UC patients. The difference between two groups was analysed by the Mann–Whitney *U* nonparametric test. The horizontal lines represent the median values. The potential correlations among the levels of serum immunoglobulin, the values of clinical parameters and the numbers of different subsets of memory B cells and plasmablasts were analysed by the Spearman correlation tests. BH correction was performed for multiple correlation.

To determine the potential role of different types of immunoglobulin in the pathogenesis of UC, we characterized the levels of serum IgG, IgM and IgA, and we found that the concentrations of serum IgG were significantly higher in the patients than in HC (*P* < 0.001, Fig. 5A), while there was no significant difference in the concentrations of IgM and IgA between the UC patients and HC (Fig. 5A). These data indicated that IgG may participate in the pathogenesis of UC. Further analysis indicated that the values of Mayo clinic score, and the concentrations of serum CRP, but not the values of ESR, were positively correlated with the concentrations of IgG (*P* = 0.024, *r* = 0.546; *P* = 0.024, *r* = 0.557, Fig. 5B). In addition, the concentrations of IgG were negatively associated with the numbers of IgG^+^ IgD^−^ CD27^+^ CD19^+^ memory B cells, and positively associated with the numbers of CD138^+^ CD38^+^ CD20^−^ CD19^+^, and IgG^+^ CD38^+^ CD20^−^ CD19^+^ plasmablasts (*P* = 0.034, *r* = −0.446; *P* = 0.015, *r* = 0.562; *P* = 0.026, *r* = 0.479, Fig. 5C). There was no significant association between the numbers of CD20^−^ CD19^+^ plasmablasts and the concentrations of IgG in the UC patients (data not shown). Collectively, our data suggest that significantly increased concentrations of serum IgG may be associated with decreased frequency of IgG^+^ IgD^−^ CD27^+^ CD19^+^ memory B cells and increased CD19^+^ CD20^−^ plasmablasts during the development of UC, and involved in the dysregulated immune response during the process of UC.

### The values of clinical parameters, numbers of IgG^+^ IgD^−^ CD27^+^ CD19^+^ memory B cells and plasmablasts in UC patients following treatment with mesalazine

**Figure 6:**
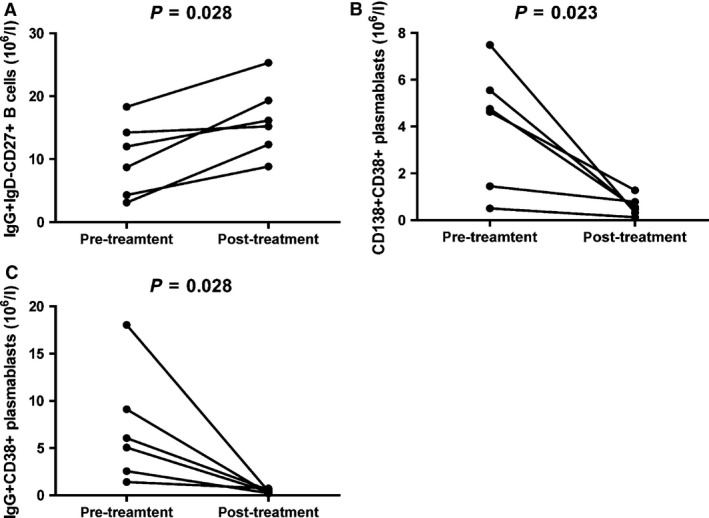
Altered numbers of IgG^+^ IgD^−^ >CD27^+^ memory B cells, CD138^+^ and IgG^+^ plasmablasts in UC patients after treatment with mesalazine. The numbers of IgG^+^ IgD^−^ CD27^+^ memory B cells and plasmablasts were compared in UC patients pre‐ and post‐treatment. Data are expressed as the real numbers of B cells or plasmablasts of individual subjects (*n* = 6 for **A**–**C**). The difference between pre‐ and post‐treatment patients was analysed by the Wilcoxon test. (**A**) The numbers of IgG^+^ IgD^−^ CD27^+^ memory B cells in individual patients pre‐ and post‐treatment. (**B** and **C**) The numbers of CD138^+^ CD38^+^ and IgG^+^ CD38^+^ plasmablasts in individual patients pre‐ and post‐treatment.

Next, we examined the impact of treatment with mesalazine on the values of clinical parameters, the numbers of circulating IgG^+^ IgD^−^ CD27^+^ CD19^+^ memory B cells and plasmablasts in UC patients, who were followed up for 8–12 weeks. There were altogether six patients with complete records and the other 17 patients failed to follow‐up. The concentrations of serum CRP and the values of ESR decreased, while the levels of haemoglobin, hematocrit and serum albumin increased significantly. There was no significant difference in the values of other clinical parameters (Table 2). Characterization of circulating memory B cells and plasmablasts revealed that the numbers of circulating IgG^+^ IgD^−^ CD27^+^ CD19^+^ memory B cells in patients after treatment were significantly greater than that in those before treatment (*P* = 0.028, Fig. 6A). Furthermore, the numbers of CD138^+^ CD38^+^, and IgG^+^ CD138^+^ CD38^+^ plasmablasts in the patients after treatment were significantly less than in those before treatment (*P* = 0.023, Fig. 6B; *P* = 0.028, Fig. 6C). There was no significant difference in the numbers of IgG^+^ IgD^−^ CD27^+^ CD19^+^ memory B cells and plasmablasts between the patients after treatment and HC (data not shown). Collectively, treatment significantly reduced inflammation and anaemia, accompanied by increasing the numbers of IgG^+^ IgD^−^ CD27^+^ CD19^+^ memory B cells and decreasing the numbers of plasmablasts in UC patients.

**Table 2:** The effect of treatment on the values of clinical measures in the follow‐up UC patients

Parameters	Pre‐treatment	Post‐treatment
Age (years)	46 (22–64)	46 (22–64)
Gender: female/male	2/4	2/4
WBC (10^9^/l)	7.80 (4.02–10.35)	6.54 (4.98–9.32)
Lymphocytes (10^9^/l)	2.07 (0.92–3.39)	1.91 (0.53–3.95)
ESR (mm/hr)	25 (18–56)^a^	6.5 (3–18)
CRP (mg/l)	32.5 (7.2–55.1)^a^	3.8 (2.0–5.9)
Haemoglobin (g/l)	103 (85–134)^a^	142 (124–167)
Hematocrit (%)	36.4 (29.2–44.3)^a^	44.7 (39.5–51.0)
Serum albumin (g/l)	34.25 (25.46–40.30)^a^	43.10 (39.07–48.92)
Mayo clinic score	8 (5–12)^a^	3 (2–4)

a *P* < 0.05 *versus* the post‐treatment.

Data are median (range) or real case number.

Normal values: WBC: 3.50–9.50 (10^9^/l), Lymphocytes: 1.10–3.20 (10^9^/l), ESR: 0–15 (mm/hr), CRP: 0–3 (mg/l), Haemoglobin: 130–175 (g/l), Hematocrit: 40.0–50.0 (%), and Serum albumin: 40.00–55.00 (g/l).

WBC: White blood cell counts; ESR: Erythrocyte sedimentation rate; CRP: C‐reactive protein; NA: Not available.

## Discussion

Dysregulated frequency of different subsets of memory B and plasma cells has been associated with the pathogenic process of many inflammatory diseases in humans 15, 16, 19, 20, 21, and humoural responses enhanced by T helper type 2 cell‐dominated immunity have been implicated in the UC pathogenesis 28. This study characterized the roles of different subsets of circulating memory B cells, plasmablasts, as well as the levels of serum immunoglobulin in UC patients and HC. We found that the decreased numbers of IgG^+^ IgD^−^ CD27^+^ CD19^+^ memory B cells were correlated negatively with increased levels of serum IgG and CD20^−^ CD19^+^ plasmablasts in the UC patients.

Memory B cells are CD27^+^ and can rapidly respond to antigen and generate immunoglobulin during secondary immune responses 29. In this study, we found that the numbers of IgD^+^ CD27^−^ CD19^+^ naïve B cells in the UC patients were significantly greater than that in the HC, while the numbers of IgD^+^ CD27^+^ CD19^+^ non‐class‐switched memory B cells in the UC patients were significantly less than that in the HC. However, we did not find significant association between the numbers of these two subsets of B cells and the values of clinic pathological features. These data suggest that inflammation reaction may promote the development and redistribution of these two subsets of B cells. Alternatively, these two subsets of B cells may not play a major role in the pathogenesis of UC. We characterized the numbers of circulating IgD^−^ CD27^+^ CD19^+^ class‐switched memory B cells and found no significant difference in the numbers of class‐switched memory B cells between the UC patients and HC, consistent with previous observations 15, 16, 30. Further analysis revealed that the percentages of IgG^+^ IgD^−^ CD27^+^ CD19^+^, but not IgM^+^ IgD^−^ CD27^+^ CD19^+^, memory B cells in the UC patients were significantly lower than that in the HC. More interestingly, the values of Mayo clinic score and ESR as well as the levels of serum IgG were correlated negatively with the numbers of IgG^+^ IgD^−^ CD27^+^ CD19^+^ memory B cells in the patients. It is possible that antigen may activate memory B cells, which differentiate into plasma cells, leading to antigen‐specific IgG production and the pathogenesis of UC. Therefore, IgG^+^ IgD^−^ CD27^+^ CD19^+^ class‐switched memory B cells are exhausted and may be a sensitive marker for evaluating of plasma cells. Collectively, our data suggest that the decreased numbers of IgG^+^ IgD^−^ CD27^+^ CD19^+^ memory B cells may be associated with the development of UC in Chinese.

Memory B cells are activated by antigens and differentiate into plasma cells, which then migrate into the bone marrow and lymphoid tissues, where they secrete immunoglobulin 31. We further investigated the role of circulating plasmablasts in the development of UC, and found that the numbers and percentages of CD20^−^ CD19^+^, CD138^+^ CD38^+^ CD20^−^ CD19^+^, and IgG^+^ CD38^+^ CD20^−^ CD19^+^ plasmablasts in the UC patients were significantly higher than that in the HC, and positively correlated with the values of Mayo clinic score and CRP, or ESR in the patients. Our data were consistent with previous reports that the frequency of circulating total plasmablasts is elevated in both adults 20 and children UC 21. Furthermore, our findings were consistent with previous observations of many IgG plasma cells in the biopsied colonic mucosal tissues from patients with UC, suggesting the importance of B cells in the pathogenesis of UC 32, 33, 34, 35. In addition, the numbers of CD138^+^ CD38^+^ CD20^−^ CD19^+^, and IgG^+^ CD38^+^ CD20^−^ CD19^+^ plasmablasts were correlated negatively with the numbers of IgG^+^ IgD^−^ CD27^+^ CD19^+^ memory B cells in the UC patients. Given that circulating plasmablasts are immature precursors of tissue plasma cells 36, 37, 38, we hypothesize that IgG^+^ IgD^−^ CD27^+^ CD19^+^ class‐switched memory B cells may play an important role in the pathogenesis of UC by the generation of CD138^+^ CD38^+^ CD20^−^ CD19^+^ and IgG^+^ CD38^+^ CD20^−^ CD19^+^ plasmablasts, which may infiltrate into the inflamed colonic mucosa as plasma cells 32, 33, 34.

Antigen‐specific antibodies are involved in the pathogenesis of many disorders, including UC 34, 39, 40. We characterized the levels of serum IgG, IgM and IgA in the UC patients and HC. We found that the concentrations of IgG, but not IgM and IgA, in the patients were significantly higher than that in the HC and correlated positively with the values of Mayo clinic score and the concentrations of serum CRP. Although the IgA deficiency, especially for the lack of secretory component in UC 41, 42, 43 or CD 44 has been reported in several UC cases, we did not observe such deficiency in those 23 UC patients in this population. The difference may stem from a small group of patients we studied. We will further study IgA deficiency in more Chinese patients. Our data suggest that higher levels of IgG may be involved in the pathogenesis of UC, consist with previous reports 23, 45, 46. In addition, the concentrations of serum IgG were positively associated with the numbers of CD138^+^ CD38^+^ CD20^−^ CD19^+^ and IgG^+^ CD38^+^ CD20^−^ CD19^+^ plasmablasts in the patients. Collectively, our findings suggest that increased numbers of CD20^−^ CD19^+^ plasmablasts and higher levels of serum IgG may be associated with the clinic activity of UC. Therefore, the numbers of circulating plasmablasts and serum IgG may be indicative of the disease activity in patients with UC.

Treatment of UC is challenging because of the complexity of UC pathogenesis, the variable responses to treatment and the course of the disease. 5‐Aminosalicylic acid or mesalazine is widely used for treatment of UC, especially for maintenance of remission. 5‐Aminosalicylic acid acts on epithelial cells to moderate the release of lipid mediators, inflammatory cells, cytokines and others 4. However, the mechanisms underlying the action of these drugs are far from completely understood. We found that treatment with mesalazine for 8–12 weeks not only significantly increased the numbers of IgG^+^ IgD^−^ CD27^+^ CD19^+^ memory B cells, but also decreased the numbers of CD138^+^ CD38^+^ and IgG^+^ CD38^+^ plasmablasts, which further suggest that these cells may participate in the pathogenic process of UC and be used for monitoring disease activity. We are interested in further investigating the molecular mechanisms underlying the action of these treatments in regulating memory B cells and plasmablasts in UC patients.

In summary, our data indicated significantly reduced numbers of IgG^+^ IgD^−^ CD27^+^ CD19^+^ class‐switched memory B cells, but increased numbers of CD138^+^ CD38^+^ and IgG^+^ CD38^+^ plasmablasts, and higher levels of serum IgG in the UC patients. The values of clinic parameters were negatively correlated with the numbers of IgG^+^ IgD^−^ CD27^+^ CD19^+^ memory B cells, while positively correlated with the numbers of CD138^+^ CD38^+^ and IgG^+^ CD38^+^ plasmablasts. Furthermore, these subsets of plasmablasts were negatively associated with the numbers of IgG^+^ IgD^−^ CD27^+^ memory B cells. Our findings suggest that the decreased numbers of IgG^+^ IgD^−^ CD27^+^ CD19^+^ memory B cells may already differentiate into CD138^+^ CD38^+^ CD20^−^ CD19^+^ and IgG^+^ CD38^+^ CD20^−^ CD19^+^ plasmablasts. These IgG^+^ IgD^−^ CD27^+^ CD19^+^ memory B cells and CD20^−^ CD19^+^ plasmablasts may contribute to the pathogenesis of UC, and characterization of these cells may provide a complementary approach to monitor the disease activity or therapeutic efficacy in active UC patients.

We recognized that our study had limitations, such as a relative small sample size and the lack of functional study of memory B cells and plasmablasts in the pathogenic process of UC. We are also interested in further investigating the values of these subsets of cells in the colon lesion to understand their roles in the pathogenesis of UC.

## Conflicts of interest

The authors declare no financial or commercial conflicts of interest.
